# Attachment Insecurity in Rats Subjected to Maternal Separation and Early Weaning: Sex Differences

**DOI:** 10.3389/fnbeh.2021.637678

**Published:** 2021-04-07

**Authors:** Haiyan Zeng, Zijia Yu, Qingjun Huang, Haiyun Xu

**Affiliations:** ^1^The Mental Health Center, Shantou University Medical College, Shantou, China; ^2^Xianyue Hospital/Xiamen Mental Health Center, Xiamen, China; ^3^The School of Psychiatry, Wenzhou Medical University, Wenzhou, China

**Keywords:** attachment anxiety, attachment avoidance, maternal separation, early weaning, rat, sex differences

## Abstract

Attachment insecurity in the forms of attachment anxiety and avoidance is associated with mental disorders in humans. In this research field, rodents, especially mice and rats, are commonly used to study social behaviors and underlying biological mechanisms due to their pronounced sociability. However, quantitative assessment of attachment security/insecurity in rodents has been a major challenge. The present study identified attachment insecurity behaviors in rats subjected to maternal separation (MS) during postnatal days (PD) 2–16 and early weaning (EW) during PD 17–21. This MSEW procedure has been used to mimic early life neglect in humans. After MSEW, rats continued to survive until early adulthood when they were subjected to open-field, social interaction, and elevated-plus maze tests. Compared to CNT rats in either gender, MSEW rats moved longer distances at higher velocities in the open-field. The MSEW rats also showed lower ratios of travel distance at central zone over that on whole arena of the open-field compared to CNT rats. In social interaction test, male CNT rats preferred to investigate an empty cage than females; whereas female CNT rats spent more time with a partner-containing cage as compared to males. This gender-specific difference was reversed in MSEW rats. On elevated-plus maze female CNT rats exhibited more risk-taking behaviors as compared to male counterparts. Moreover, female MSEW rats experienced a greater difficulty in making a decision on whether approaching to or averting from which arms of elevated-plus maze. Taken together, male MSEW rats behaved like attachment anxiety while females’ phenotype is alike to attachment avoidance described in humans. These results shall prompt further application of MSEW rat in abnormal psychology and biological psychiatry research.

## Introduction

Attachment refers to a selective and enduring bond between individuals including romantic attachment between adults and infant–caregiver attachment. In the latter scenario, attachment describes a complex and highly specific bond established between an infant and his/her caregiver (Bowlby, 1982). There is increasing evidence that quality of care affects emotionality and emotion regulation throughout the life course (Waters et al., 2000). It was reported that individuals reared in institutional settings exhibited deficits in emotion regulation, attachment to primary caregivers, and cognitive development (O’Connor et al., 2003; Kreppner et al., 2007; Zeanah et al., 2009; Tottenham et al., 2010). A stable sense of attachment security results from interactions with attachment figures who are available in times of need, sensitive and responsive to bids for proximity and support (Bowlby, 1973). With a secure attachment, a person tends to have a high level of self-esteem, self-stability and satisfaction as it facilitates emotion regulation and enhances affiliative behaviors between peers (Canterberry and Gillath, 2013). In contrast, insecure attachment is likely due to having an unresponsive, rejecting, inconsistent, or insensitive caretaker (Ainsworth and Bell, 1970). Clinical studies have shown that attachment insecurity is associated with some of mental health problems including depression (Catanzaro and Wei, 2020), anxiety (Bosmans et al., 2020), obsessive-compulsive disorder (Doron et al., 2012), post-traumatic stress disorder (Ein-Dor et al., 2010), suicidal tendencies (Gormley and McNiel, 2010), and eating disorders (Illing et al., 2010).

A person’s sense of attachment security is reflected by his/her location in the two-dimensional conceptual space defined by attachment anxiety and avoidance (Mikulincer and Shaver, 2007). People with low scores on these two dimensions generally feel secure and tend to employ constructive and effective affect-regulation strategies; whereas those with high score on either the attachment anxiety or avoidance dimension (or both) often have a sense of insecurity and tend to rely on secondary attachment strategies (either deactivating or hyperactivating their attachment system) to cope with threats (Cassidy and Kobak, 1988). In clinical and research practice, adult attachment style can be assessed using several self-report instruments, such as the Experiences in Close Relationships (Brennan et al., 1998), the Attachment Style Questionnaire (Hazan and Shaver, 1987), and the Relationship Questionnaire (Bartholomew and Horowitz, 1991).

Most psychological scholars concede that the core human psyche is a product of biological evolution resulting from natural selection (Panksepp, 2006). In line with this consensus, it is believed that many other animals also have emotional feelings, including anger, fear, maternal care, separation distress, social bonding, and playfulness (Panksepp, 1998, 2005). Indeed, animal studies including those on imprinting in birds (Bateson, 1966), early olfactory learning in rabbits (Hudson, 1993), and the development of affectional bonds in nonhuman primates (Harlow and Suomi, 1970) have significantly facilitated the development of attachment theory. And animal models of disrupted infant–caregiver relationship have been used to investigate the neurobiology of infant attachment and fear, as well as the maturation of emotion circuits (Callaghan et al., 2014). Particularly, adolescent and adult rats that had received less maternal care or unpredictable shock during infancy expressed anxiety-like behaviors and heightened stress responses (Macrì et al., 2008; O’Mahony et al., 2009; Sarro et al., 2014). Moreover, parental separation was shown to enhance active avoidance learning in juvenile rodents (Abraham and Gruss, 2010) while early life handling enhanced contextual conditioning in P18 rats (Beane et al., 2002). These previous findings support the view that translational models of disrupted infant–caregiver relationship are critical in understanding mental health trajectories in humans.

Different from human studies that assess human attachment style using several self-report instruments as reviewed above (Hazan and Shaver, 1987; Bartholomew and Horowitz, 1991; Brennan et al., 1998), quantitative assessment of attachment security/insecurity in animals has been a major challenge. In trying to circumvent this challenge, this animal study employed the laboratory Sprague-Dawley (S-D) rat, an ideal subject for studies of maternal care (Numan, 1994), and adapted a paradigm of maternal separation and early weaning (MSEW), which was initially designed by George et al. (2010) for mice. This paradigm has been used to mimic early life neglect in humans and is believed to influence brain development and consequently bring forth a predisposition toward mental and behavioral disorders (Carlyle et al., 2012; Strüber et al., 2014). After MSEW, rats continued to survive into early adulthood and then subjected to open-field, social interaction, and elevated-plus maze tests. The three behavioral tests have been used to estimate the explorative activity and anxiety level (Hiroi and Neumaier, 2009), social behavior (Smolensky et al., 2019), and risk-taking/anxiety-like behavior (Tillmann and Wegener, 2019) in rats, respectively. Compared to controls, MSEW rats showed higher anxiety level and social behavior deficiency in open-field and social interaction tests, as well as a risk-taking behavior on the elevated-plus maze. These behavioral abnormalities were not reported in previous studies that either applied maternal separation (MS) (Park et al., 2018; Isobe and Kawaguchi, 2019; Ströher et al., 2019) or early weaning (EW) to rats (Kanari et al., 2005; Ito et al., 2006; Shimozuru et al., 2007). In the previous studies that applied MSEW paradigm to mice (George et al., 2010; Carlyle et al., 2012), different behavioral tests were employed thus did not result in the same results as what reported in this study. Moreover, female rats were included in this study given that females were frequently overlooked in previous preclinical research due to the concern that female reproductive cycle would lead to behavioral variance in subjects. This addition allowed us to compare behavioral abnormalities in male and female MSEW rats. Interestingly, male MSEW rats behaved like attachment anxiety while females’ phenotype is alike to attachment avoidance described in humans.

## Materials and Methods

### Animals

Female S-D rats at gestational week 2 were purchased from the animal center of the Southern Medical University (Guangzhou, China) and housed in an air-conditioned room at the vivarium of Shantou University Medical College. The animals had free accesses to food and water in the room with controlled temperature in the range of 23 ± 1°C and a 12:12 h light cycle. The delivery day was defined as PD 0. An even number (with equal number in male and females) up to ten pups of each litter and their dam were culled for the next MSEW procedure or being used as controls. All animal handling and use were carried out in accordance with the guidelines set up by the Animal Care and Use Committee of Shantou University Medical College and approved by the committee.

### MSEW Procedure

**FIGURE 1:**
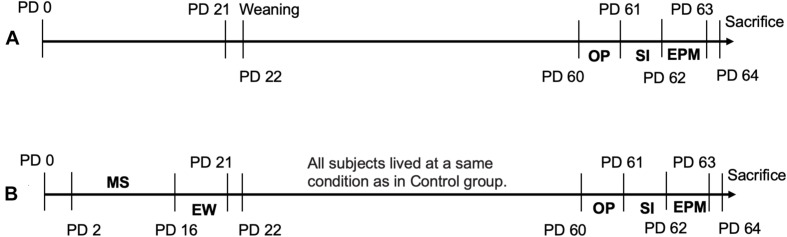
Experimental schedule and MSEW paradigm. **(A)** Shows the experimental schedule set up for rats in Control group. **(B)** Shows the MSEW paradigm and experimental schedule for rats in MSEW group. EPM, elevated plus maze test; EW, early weaning; MS, maternal separation; OP, open field test; PD, postnatal day; SI, social interaction test.

The maternal separation (MS) started on PD 2, by removing a pup from his/her dam and placing the pup in a small carton (10 × 9 × 9 cm) for 4 h per day during PDs 2–5, and 6 h per day during PDs 6–16. The MS duration increased with age because the younger the pups, the more susceptible to starvation as demonstrated in our primary experiment, in which MS for 6 h per day during PDs 2–5 led 50% of pups to die. During the separation period, which started at the same time (8:00 am) every day, pups in cartons (one pup per carton) were kept at an infant incubator (YP-100; Ningbo David Medical Device Co., Ltd., Ningbo, China) which was kept well ventilated at a controlled temperature (34°C during PDs 2–5, 32°C during PDs 6–9, 30°C during PDs 10–14, and 28°C during PDs 15–16) and a constant humidity (60%) under the light condition of 20 lux at a room of 3.5 × 4.5 m. Before and after MS, all pups in the MSEW groups (*n* = 20/group in either sex) were brought back to the cage where their dam was living, but the maternal behaviors were not monitored during the reunion period. Early weaning (EW) occurred on PD 17 when a home-made soft diet (powdered rodent chow in tap water) was provided to the pups kept at cartons (one pup per carton). Starting at PD 22, the MSEW rats of a same litter were housed in group (5 pups/cage, 485 × 350 × 200 mm) by sex. The pups in Control groups (*n* = 20/group in either sex male) were raised by their dams under the standard laboratory condition as described above and weaning started at PD 22. The body weight of all pups was weighed at PD 7, 14, 21, and 30, respectively. The schematic diagram of above procedures was shown in Figure 1. Nothing was done to control the estrous circle of females as the MSEW procedure was applied to immature rats in this study (rats take about 3 weeks to mature and begin fending for themselves). And meta-analyses have shown that naturally cycling female mice and rats present no more variance in broadly categorized behavioral measures than males (Prendergast et al., 2014; Becker et al., 2016; Beery, 2018).

### Behavioral Tests

The behavioral tests carried out in this study include open-field test, social interaction test, and elevated-plus maze test. They were administered during PDs 60–62, once a day in the order of increasing aversiveness to minimize the impact of immediate behavioral testing on subsequent tests. Before the commencement of behavioral tests, rats were transported to the testing room (about 10 square meter size) and stayed there overnight for adaptation.

#### Open-Field Test

The wooden open field box (100 × 100 × 60 cm) was painted in black and sheltered by a blue drape in the behavioral test room, which was lighted with three white fluorescents (in a total of 15 lux) placed 160 cm above the arena. Each individual rat was placed in the center of the open-field box and allowed to move freely for 12 min. The first 2 min were defined as the adaptation period and the data from this period was not included for analysis. A video tracking system (EthoVision XT 9.0; Noldus Information Technology, Wageningen, Netherlands) was used to monitor the tested rat. For each tested rat, the moving distances on the whole arena (TD) and its central zone (CD, the central part of 50 × 50 cm), and time spent on the central zone (CT) were recorded. The ratio of CD/TD was calculated. The TD was considered an index of locomotor activity and CD/TD index of anxiety level. In addition, the moving velocity (MV) of rats in the open-field was also calculated. The floor and inner walls of the box were cleaned with 70% ethanol after each test.

#### Social Interaction Test

This test was carried out in the same open-field box lighted by the same white fluorescents as in the open-field test. It consists of two sessions and an interval between sessions. Each session persisted for 150 second (S) while the interval persisted for 1 min thus the whole test persisted for 6 min as described previously (Challis et al., 2013). The procedure was also successfully employed in the other animal studies that measured social behaviors of rodents (Krishnan et al., 2007; Browne et al., 2018; Zhang et al., 2018). Before the test, all rats were housed in group (5 rats/cage) as mentioned above. During the first session, an empty (E session) wire mesh cage (12 × 12 × 18 cm) was placed at one end of the open-field arena (100 × 100 cm) where a tested rat was allowed to move freely. During the second session, the conditions were identical except that an unfamiliar conspecific partner (C session) had been introduced into the cage before a tested rat was placed in the open-field box. The partner was matched with the tested rat in gender, age, and body weight, but they were neither littermates nor cage mates. Between the two test sessions, the tested rat was removed from the box and placed back into his/her home cage for 60 S. The video tracking system was used to monitor the tested rat. The time spent by the tested rat at the interaction zone (a 16-cm-wide corridor around the cage) was recorded.

#### Elevated-Plus Maze Test

The elevated-plus maze consists of four radial arms (two closed, 50 × 10 × 40 cm; two open, 50 × 10 × 2 cm) elevated 60 cm above the floor. Under the same lighting condition as that in the open-field test, rat was placed at the central junction, facing a closed arm, and the activity of the rat on the elevated-plus maze was recorded during the subsequent 10 min. The first 2 min were defined as the adaptation period and the performance of the rat in the remaining 8 min was analyzed. The time spent by a tested rat on the central junction (Tcj), open (To) and closed arms (Tc), and the number of entries to these locations (Ncj, No, and Nc) were recorded. The ratio of To/Tc was calculated and considered an index of anxiety level. In the preliminary experiment, MSEW rats spent much more time on open arms of the elevated-plus maze compared to CNT rats. We speculated that this abnormal behavior in MSEW rats was indicative of a risk-taking behavior instead of an anxiolytic effect induced by the paradigm. In order to confirm and further interpret this abnormal behavior, we elongated the test time from the standardized 5 to 8 min and included Tcj and Ncj for data analysis.

### Statistical Analysis

SPSS17.0 (IBM Corp., Armonk, NY, United States) was used to analyze all the data which were expressed as mean ± SD. The Shapiro–Wilk test was used to test the data for normality. For social interaction data, independent paired *t* tests were done to compare mean values from E and C sessions of a same group (CNT or MSEW), and from CNT and MSEW groups in a same E or C session. For the other data, two-way ANOVA was done before post-hoc comparisons (*F*-test). The significant threshold was set at 0.05.

## Results

### The Weight Gain of Rats and Effect of MSEW

**FIGURE 2:**
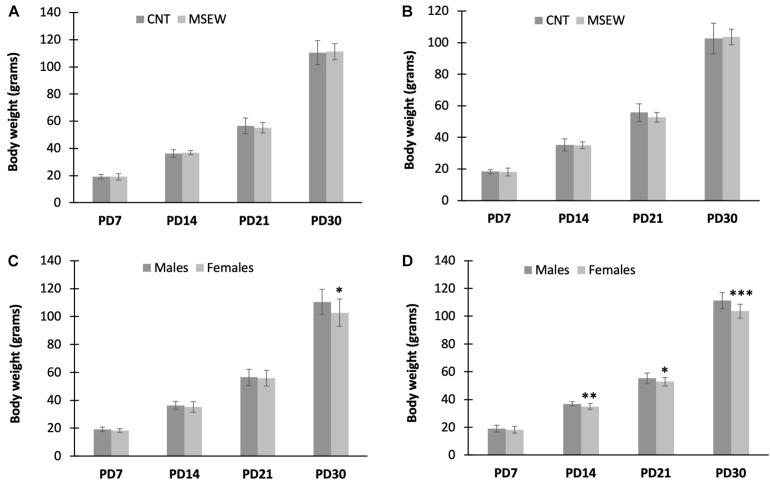
Body weight of rats measured at four postnatal time points. **(A)** Body weight of male rats in CNT and MSEW groups. **(B)** Body weight of female rats in CNT and MSEW groups. **(C)** Body weight of male and female CNT rats. **(D)** Body weight of male and female MSEW rats. Data were expressed as mean ± SD. *n* = 20/group.

Infant rats rely on attaching to his/her dam for care and nourishment. MSEW may exert significant impacts on rat pups in respect of physiological and psychological parameters. We wanted to establish a reliable MSEW paradigm that has no or a minimum effect on physiological parameters of subjects. In preliminary experiments, MS lasted for 6 h/day during PD 2–5 and 8 h/day during PD 6–16. This protocol led to a high fatality (about 50%) in MSEW rats during the MS period. As such, the procedure was modified as reported here, i.e., 4 h/day during PD 2–5 and 6 h/day during PD 6–16. This modified procedure caused no rat death. The data of body weight measured at PD 7, 14, 21, and 30 were analyzed by two-way ANOVA. For male rats, two-way ANOVA showed (1) no significant interaction between treatment and time (*F*_(__3_,_159__)_ = 0.558, *p* = 0.644), (2) a significant effect of measuring time on body weight of rat pups (*F*_(3,__159__)_ = 2,813.101, *p* = 0.000), i.e., the body weight of rat pups increased with age, (3) MSEW showed no effect on weight gain of rat pups (*F*_(1,159)_ = 0.046, *p* = 0.831) (Figure 2A). Similar results were found in female rats, i.e., there was no significant interaction between time and treatment (*F*_(3,159)_ = 0.939, *p* = 0.423), the body weight of female pups increased with age (*F*_(3,159)_ = 2,261.789, *p* = 0.000), but MSEW had no effect on weight gain (*F*_(1,159)_ = 0.483, *p* = 0.488) (Figure 2B).

In addition, another two-way ANOVA was carried out with gender and measuring time as two main factors. The results showed significant interactions between gender and measuring time in both CNT (*F*_(3,159)_ = 3.588, *p* = 0.015) and MSEW (*F*_(3,159)_ = 7.025, *p* < 0.001) rats. Both gender (*F*_(1,159)_ = 8.083, *p* = 0.005) and measuring time (*F*_(3,159)_ = 1,746.947, *p* < 0.000) had significant effects on body weight of rat pups in CNT and MSEW groups. Post-hoc comparisons showed that male CNT rats were heavier than females at PD 30 (Figure 2C). As for MSEW rats, females had lower body weight than males at PD 14 and thereafter (Figure 2D).

### Effects of MSEW on the Performance of Rats in Open-Field Test

**FIGURE 3:**
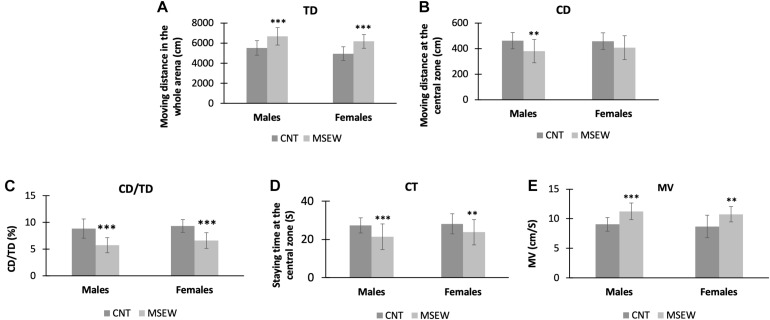
Performance of rats in the open field test. **(A)** Moving distances of CNT and MSEW rats on the whole arena of open field. **(B)** Moving distances of CNT and MSEW rats at the central zone of open field. **(C)** The values of CD/TD of CNT and MSEW rats. **(D)** The staying time of rats at the central zone of open field. **(E)** The moving velocities of rats in the open field. Data were expressed as mean ± SD. *n* = 15/group. **p < 0.01, ***p < 0.001, MSEW vs Control.

In the open-field test, we analyzed the parameters TD, CD, CD/TD, CT, and MV as shown in Table 1. Both males and females in either CNT or MSEW rats showed comparable performances in terms of the parameters mentioned above. But differences were obvious between CNT and MSEW groups in either males or females. Specifically, two-way ANOVA revealed that there was no interaction (*F*_(1, 59)_ = 0.113, *p* = 0.738) between gender and treatment in regard of TD, but each of the main factors had a significant effect (treatment, *F*_(1, 59)_ = 44.539, *p* = 0.000; gender, *F*_(1, 59)_ = 5.141, *p* = 0.027) on this parameter. Post-hoc comparisons indicated that male and female MSEW rats moved longer TDs compared to CNT groups (Figure 3A), but no difference between males and females in both CNT and MSEW rats. As for CD, there was no interaction (*F*_(1, 59)_ = 0.557, *p* = 0.458) between the two main factors. Treatment (*F*_(1, 59)_ = 10.199, *p* = 0.002), but not gender (*F*_(1, 59)_ = 0.338, *p* = 0.563), exerted a significant effect on this parameter. Post-hoc comparisons indicated that male MSEW rats had a shorter CD compared to male CNT group (Figure 3B). In regard of CD/TD, there was no interaction (*F*_(1, 59)_ = 0.272, *p* = 0.604) between the two main factors. Treatment (*F*_(1, 59)_ = 57.377, *p* = 0.000), but not gender (*F*_(1, 59)_ = 3.277, *p* = 0.076), had a significant effect on this parameter. Post-hoc comparisons indicated that both male and female MSEW rats had lower values of CD/TD compared to CNT groups (Figure 3C). For CT, there was no interaction (*F*_(1, 59)_ = 0.286, *p* = 0.595) between the two main factors. Treatment (*F*_(1, 59)_ = 12.147, *p* = 0.001), but not gender (*F*_(1, 59)_ = 1.162, *p* = 0.286), had a significant effect. Post-hoc comparisons indicated that both male and female MSEW rats spent less time at the central zone compared to CNT groups (Figure 3D). In regard of MV, there was no interaction (*F*_(1, 59)_ = 0.025, *p* = 0.874) between the two main factors. Treatment (*F*_(1, 59)_ = 32.269, *p* = 0.000), but not gender (*F*_(1, 59)_ = 1.295, *p* = 0.260), had a significant effect. Post-hoc comparisons indicated that both male and female MSEW rats moved faster in the open-field compared to CNT groups (Figure 3E). In summary, MSEW increased anxiety levels in either male or female rats, there was no sex difference in this regard.

**TABLE 1:** Performance of adult rats in open-field test.

	**CNT**		**MSEW**	
**Measurements**	**Males**	**Females**	**Males**	**Females**
TD (cm)	5,331.589 (186.64)	4,958.63 (178.45)	6,685.50 (225.70)***	6,182.62 (177.74)***
CD (cm)	462.46 (16.54)	459.03 (16.94)	380.61 (24.29)**	408.20 (23.99)
CD/TD (%)	8.83 (1.79)	9.32 (1.21)	5.73 (1.42)***	6.62 (1.46)***
CT (S)	27.21 (1.02)	28.11 (1.36)	21.37 (1.73)***	23.75 (1.69)**
MV (cm/S)	9.04 (1.17)	8.67 (1.89)	11.24 (1.41)***	10.75 (1.29)***

*Data were expressed as means (SD) (*n* = 15).*

CNT, rats in this group were raised under normal condition with no experience of MSEW; MSEW, rats in this group were subjected to MSEW procedure; CD, distance traveled at central zone of open-field; TD, distance traveled on the whole arena of open-field; CT, time spent on the central zone; MV, moving velocity of rats in the open-field.

**p < 0.01, ***p < 0.001, MSEW rats vs CNT rats in the same gender.

### Gender-Specific Performance of Rats in Social Interaction Test: Effects of MSEW

**FIGURE 4:**
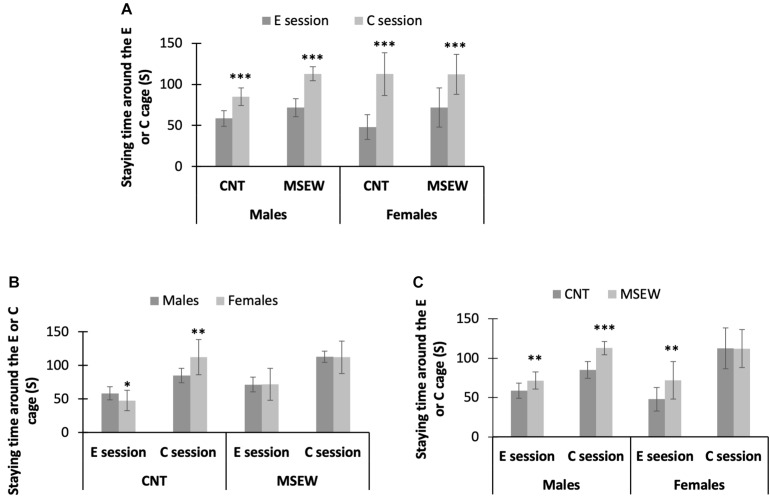
Performance of rats in social interaction test. **(A)** The comparisons between the E session and C session, in terms of the time spent on interaction zone. **(B)** The comparisons between males and females in either CNT or MSEW rats, in terms of the time spent on interaction zone. **(C)** The comparisons between CNT and MSEW rats in either gender, in terms of the time spent on interaction zone. Data were expressed as mean ± SD. n = 15/group. *p < 0.05, **p < 0.01, ***p < 0.001.

We focused on the time spent by rats at the social interaction zone around a wire mesh cage without or with an unfamiliar conspecific in the social interaction test. All data are shown in Table 2. First, all male and female rats in both CNT and MSEW groups spent much more time at the social interaction zone during the C session relative to E session (Figure 4A), confirming the presence of social preference of CNT rats, i.e., preference to investigate a novel conspecific over a novel object. This social play function keeps working in MSEW rats. Second, male CNT rats spent more time around an empty cage relative to females, suggesting that males preferred to investigate a novel object than females. In contrast, female CNT rats spent more time at the interaction zone in the presence of an unfamiliar conspecific in the cage compared to males, suggesting that females preferred to investigate a novel conspecific. These sex differences, however, were not seen between male and female MSEW rats (Figure 4B), suggesting that MSEW exerted different effects on the social behaviors of male and female rats. Third, male MSEW rats played for longer durations at the social interaction zone during E and C sessions as compared to controls, while female MSEW rats spent a longer duration at the social interaction zone during E session but not C session as compared to female CNT rats (Figure 4C). These results suggest that MSEW increased the social preference of male rats, but made female rats prefer to investigate a novel object (the empty cage), which may be indicative of an attachment avoidance behavior.

**TABLE 2:** Performance of adult rats in social interaction test.

**Staying time around**	**CNT**		**MSEW**	
	**Males**	**Females**	**Males**	**Females**
E cage (S)	58.60 (9.55)	47.85 (15.15)*	71.59 (10.97)	71.84 (23.80)
C cage (S)	85.08 (10.72)^##^	112.44 (15.93)**^,###^	112.88 (8.38)^##^	112.20 (24.20)^##^

Data were expressed as means (SD) (*n* = 15). CNT, rats in this group were raised under normal condition with no experience of MSEW; MSEW, rats in this group were subjected to MSEW procedure; E cage, an empty cage; C cage, a cage containing an unfamiliar conspecific. *p < 0.05, **p < 0.01, males vs females in either CNT or MSEW groups. ^##^*p* < 0.01, ^###^*p* < 0.0001. E (cage) session vs C (cage) session.

### Gender-Specific Performance of Rats in Elevated-Plus Maze Test: Effects of MSEW

**FIGURE 5:**
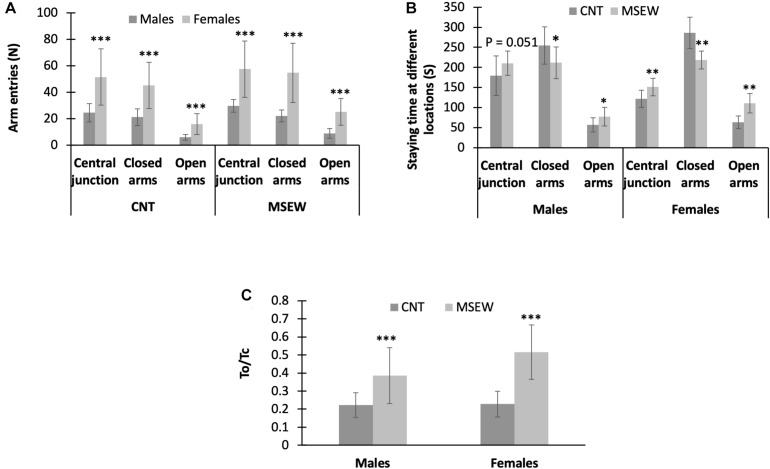
Performance of rats on elevated-plus maze. **(A)** The comparisons between males and females in either CNT or MSEW rats, in terms of the number of entries into different parts of the elevated-plus maze. **(B)** The comparisons between CNT and MSEW rats in either gender, in terms of the time spent at different parts of the elevated-plus maze. **(C)** The comparisons between CNT and MSEW rats in either gender, in terms of To/Tc ratio. Data were expressed as mean ± SD. *n* = 15/group. *p < 0.05, **p < 0.01, ***p < 0.001.

All data regarding the performance of rats on the elevated-plus maze are shown in Table 3. First, female (CNT, MSEW) rats entered open arms, closed arms, and central junction more frequently than males (Figure 5A). Second, MSEW (male, female) rats spent much more time on open arms and central junction, but less time in closed arms, as compared to CNT rats (Figure 5B). Third, two-way ANOVA showed a significant interaction between treatment and gender (*F*_(1, 59)_ = 4.248, *p* = 0.044) on values of To/Tc (%); both the treatment (*F*_(1, 59)_ = 53.932, *p* = 0.000) and gender (*F*_(1, 59)_ = 4.831, *p* = 0.032) exerted significant effects. Post-hoc comparisons showed that MSEW rats had greater values of To/Tc than CNT rats in either males or females (Figure 5C), implying that MSEW might have an anxiolytic effect on the rats. This interpretation seems to be contrary to the conclusion from open-field test, i.e., MSEW increased anxiety levels in either male or female rats.

**TABLE 3:** Performance of adult rats on the elevated-plus maze test.

	**CNT**		**MSEW**	
**Measurements**	**Males**	**Females**	**Males**	**Females**
Tcj (S)	179.39 (50.33)	121.74 (21.48)***^,#^	210.44 (30.97)	151.23 (22.73)***^,##^
Tc (S)	254.54 (47.57)	285.78 (40.22)	211.41 (40.05)^#^	218.51 (22.15)^##^
To (S)	56.77 (18.28)	63.30 (16.34)	77.38 (23.51)^#^	110.57 (25.14)***^,##^
To/Tc	0.22 (0.07)	0.23 (0.07)	0.39 (0.02)^###^	0.52 (0.02)*^,###^
Ncj (N)	24.49 (7.00)	51.50 (21.21)***	29.60 (4.76)	57.53 (21.28)***
Nc (N)	21.13 (6.30)	45.22 (17.50)***	22.09 (4.55)	54.68 (22.38)***
No (N)	5.91 (2.18)	15.88 (7.89)***	8.74 (3.75)	25.15 (10.09)***

*Data were expressed as means (SD) (*n* = 15).*

CNT, rats in this group were raised under normal condition with no experience of MSEW; MSEW, rats in this group were subjected to MSEW procedure; Tcj, staying time (in seconds) at the central junction; Tc, staying time in closed arms; To, staying time on open arms. Ncj, number of entries into the central junction; Nc, number of entries into closed arms; No, number of entries into open arms.

*p < 0.05, ***p < 0.0001, males vs females in either CNT or MSEW rats.

*^#^*p* < 0.05, ^##^*p* < 0.01, ^###^*p* < 0.0001, CNT vs MSEW rats in either males or females.*

To dissolve this conflict, we calculated values of Tcj/Ncj and To/No of all animal groups. These parameters reflect the staying time per visiting and are of help in confirming the so-called anxiolytic effect of MSEW on rats. We found that values of these two parameters in rats were not changed by MSEW, i.e., CNT and MSEW groups were comparable in terms of Tcj/Ncj and To/No (not shown). The results do not support the anxiolytic effect of MSEW.

## Discussion

This study is the first one reporting attachment-related behaviors in rats subjected to MSEW procedure during the first 3 weeks after birth. The main findings include (1) male and female MSEW rats moved longer distances on whole arena of the open-field at higher velocities and showed lower values of CD/TD compared to respective controls; (2) in the social interaction test, male CNT rats preferred to investigate a novel object than females. In contrast, female CNT rats preferred to investigate a novel conspecific compared to males. This gender-specific difference was not seen in MSEW rats. Moreover, MSEW increased the social preference of male rats, but made female rats prefer to investigate a novel object (the empty cage), which may be indicative of a social avoidance behavior (Scholl et al., 2019); (3) on elevated-plus maze, females (CNT, MSEW) rats entered open arms, closed arms, and central junction more frequently than males irrespective of MSEW experience, MSEW (males, females) rats spent much more time on open arms and central junction, but less time in closed arms, as compared to CNT rats irrespective of gender, implying an anxiolytic effect of MSEW. But values of Tcj/Ncj and To/No were comparable across all animal groups, which do not support the anxiolytic effect of this paradigm.

The present study is the first one applied the MSEW paradigm to rats while the others applied MS (Park et al., 2018; Isobe and Kawaguchi, 2019; Ströher et al., 2019) or EW (Kanari et al., 2005; Ito et al., 2006; Shimozuru et al., 2007) to rats. And a few previous studies applied MSEW procedure to mice (Carlyle et al., 2012; George et al., 2010). Long-term MS was shown to induce compensatory maternal care as seen in rat dams (Macrì et al., 2008). EW decreased play-fighting behaviors during the postweaning developmental period in Wistar rats, and increased anxiety levels during early adulthood (Shimozuru et al., 2007). In another study, EW rats showed increased locomotion and greater rearing activity in the open field but did not show anxiety increase in the open-field and elevated-plus maze tests (Ishikawa et al., 2014). MSEW mice spent less time on central part of the open-field and moved significantly faster than controls during the first 5 min of test (George et al., 2010; Carlyle et al., 2012). In line with these previous studies, MSEW rats in this study presented higher levels of anxiety demonstrated by shorter moving distance at central zone of the open-field and less time spent at the zone relative to controls. Moreover, MSEW rat moved a greater amount of distance with a faster speed on whole arena of the open-field as compared to CNT rats, indicating a higher level of locomotor activity induced by MSEW. Taken together, MSEW exerted same anxiogenic effects on male and female rats in open-field test.

In the social interaction test, both MSEW and CNT rats were able to tell an empty cage from a partner-containing cage as evidenced by spending more time at the social interaction zone in the presence of a partner-containing cage compared to the scenario of the empty cage, confirming the social preference of the rats, i.e., preference to investigate a novel conspecific over a novel object. Further analysis revealed different performance of male and female CNT rats in the social interaction test, i.e., male CNT rats spent more time with the empty cage relative to females whereas female CNT rats spent much more time with the partner-containing cage than male CNT rats did. These results suggest that male rats prefer to investigate a novel object (the empty cage) whereas females are featured with the social preference. Intriguingly, these sex-specific social behaviors are in contrast to the observation of a recent animal study in which female rats spent a greater amount of time with the novel object (empty cage) as compared to males (Scholl et al., 2019). In seeking the impact factors that may account for the contrast social behavior patterns between the rats across the two studies, we noticed a major difference between the social interaction test procedures applied in the two studies. In brief, each session of the two test sessions lasted for 5 min in the study by Scholl et al. (2019) whereas it was 2.5 min long in the present study. During a longer duration of testing, a tested rat is more likely to adapt to an environment (empty cage or the same cage with an unfamiliar conspecific). With the only two studies compared, it is hard to know which test duration is more appropriate.

More importantly, these sex-specific patterns in social behavior were not seen in MSEW rats, indicating that MSEW differently impacted the performance of male and female rats in social interaction test. Specifically, MSEW made female rats spent more time with the empty cage relative to CNT rats, that is, it reversed the social behavior pattern in CNT rats in whom male (CNT) rats spent more time with the empty cage relative to females. In either case, a preference for a novel object is indicative of a social avoidance behavior (Scholl et al., 2019). Relevantly, a previous animal study reported that parental separation enhanced active avoidance learning in juvenile rodents (Abraham and Gruss, 2010). These social avoidance behaviors in animals are reminiscent of the attachment avoidance seen in humans (Mikulincer and Shaver, 2007; Mikulincer and Shaver, 2019). People with avoidant attachment rely on deactivating strategies, i.e., do not seek proximity, deny attachment needs, and avoid closeness and interdependence in relationships (Mikulincer and Shaver, 2007).

Another significant effect of MSEW on social behaviors of rats manifested as more time spent during E and C sessions by male MSEW rats as compared to CNT rats, suggesting that MSEW increased the social preference of male rats. Along with increased anxiety level of MSEW rats as shown in open-field test, the performance of male MSEW rats in social interaction test may be interpreted as a phenotype of attachment anxiety, another type of attachment insecurity seen in humans (Mikulincer and Shaver, 2007). People with attachment anxiety rely on hyperactivating strategies demonstrated by energetic attempts to achieve proximity, support, and love as they have no confidence that these resources will be provided (Cassidy and Kobak, 1988).

On elevated-plus maze, female (CNT, MSEW) rats entered open arms, closed arms, and central junction more frequently than males, MSEW (male, female) rats spent much more time on open arms and central junction, but less time in closed arms, as compared to CNT rats. These results are in line with a recent study reporting that female rats spent more time on open arms and more frequently entered open arms as compared to males. Females also traveled a greater distance than males regardless of estrus cycle stage (Scholl et al., 2019). Moreover, this less anxiety-like behavior on the elevated-plus maze has been observed in many of previous studies of female vs. male rats (Diaz-Veliz et al., 1997; Frye et al., 2000; Aguilar et al., 2003; Lopez-Aumatell et al., 2008, 2011). It was speculated that the sex differences in rodent tests of anxiety relate to sex-differences in stress-coping as evidenced by the observation that female rats showed enhanced reactive or compensatory coping strategies to stressors as compared to males (Lopez-Aumatell et al., 2008). Moreover, females have been shown to be more vulnerable to mild stress than males exposed to the same stressors as evidenced by biological measures such as altered serotonergic activity and increased corticosterone (Dalla et al., 2005, 2011).

The aforementioned data of previous studies and this one suggest that the seemingly less anxiety-like behavior of female rats may be viewed as a different form of anxiety-like behavior that are not well captured by traditional testing. Indeed, the elevated-plus maze test was used to assess risk-taking behavior of rats (Tillmann and Wegener, 2019). From this point of view, that MSEW rats spent more time on open arms and central junction but less time in closed arms as compared to CNT rats may be interpreted as a higher level of risk-taking behavior due to an anxiogenic instead of an anxiolytic effect of this paradigm. This interpretation is in line with the inference from the open-field test data, i.e., MSEW increased anxiety levels in either male or female rats. But the sex-specific effects of MSEW on behaviors of rats on the elevated-plus maze suggest that females are more vulnerable to MSEW compared to males. Following this notion, that MSEW rats spent more time on the central junction of elevated-plus maze indicates that they experienced a greater difficulty in making a decision on which arms to approach, i.e., they could not correctly cope with the threats of staying on the elevated-plus maze. Then that MSEW rats spent more time on open arms indicates an incorrect coping strategy of them in face of these danger parts of the apparatus. Taken together, the data from elevated-plus maze test provide further evidence for MSEW-induced attachment insecurity in rats.

Supporting evidence for the adverse effects of MSEW also came from the weight gain data of rats, including (1) body weights of CNT and MSEW rats were comparable at each timepoint, (2) male CNT rats were heavier than females at PD 30, and (3) female MSEW rats had lower body weight than male MSEW rats at PD 14 and thereafter. The first finding suggests that MSEW did not result in any nutritional deficits or did not induce significant changes in feeding behavior of rats during the MSEW period. This is in accordance with the previous study by George et al. (2010), in which the MSEW protocol showed no effect on weight gain of mice during PD 10–83. The second finding is fully consistent with the weight gain chart of S-D rats, in which males and females began to differ immediately after postnatal week 4. Interestingly, the gender-specific difference in rat weight gain appeared at PD 14 and continued thereafter in MSEW rats, indicating that female rats are more sensitive to MSEW while males are more tolerable to MSEW. This interpretation is in line with the behavioral data presented above indicating higher level of risk-taking behavior and attachment avoidance phenotype in female MSEW rats as compared to male counterparts featured with attachment anxiety. More importantly, the early onset of lower weight in female MSEW rats relative to males implies that attachment avoidance hurt the female subjects more than attachment anxiety did. This inference has specific relevance to extant clinical observations pointing to a higher prevalence of affective disorders such as anxiety and depression in women (Kessler et al., 1994, 2012; Seeman, 1997; Holden, 2005; Altemus et al., 2014).

In conclusion, MSEW induced emotional dysregulation in early adult rats with behavioral phenotype alike to attachment insecurity seen in humans as a consequence of early life adversity. Specifically, the behavioral phenotype of male MSEW rats is alike to attachment anxiety as evidenced by higher anxiety level detected in open-field test and much more social interaction time in both E and C sessions in the social interaction test. The phenotype of female MSEW rats is like attachment avoidance demonstrated by higher anxiety level measured in open-field test, risk-taking behaviors on the elevated -plus maze, and preference to investigate a novel object (an empty cage) in social interaction test as compared to female CNT rats. The attachment insecurity in MSEW rats made it difficult for them to make a decision on whether approaching to or averting from which arms of the elevated-plus maze. Last but not least, the delayed weight gain in female MSEW rats relative to males implies that attachment avoidance hurt the female subjects more than attachment anxiety did. This inference has relevance to the clinical observations pointing to higher prevalence of affective disorders such as anxiety and depression in women.

We are aware of a couple of limitations of this study. For instance, the maternal care behaviors of dams following the separation period were not monitored. Previous studies have shown that neonatal social isolation alters both maternal and pup behaviors in rats (Zimmerberg et al., 2003; Starr-Phillips and Beery, 2014). Technically, further social tests would be required to provide adequate proof for the conclusions from this study. These could include mating behavior, response to socially relevant cues, i.e., USV (ultrasonic vocalizations) playback paradigms or social odor tests. In a recent study, social and non-social behaviors together with concomitant emission of 50-kHz USV were measured in rats (Redecker et al., 2019).

## Data Availability Statement

The raw data supporting the conclusions of this article will be available on request to the corresponding author.

## Ethics Statement

The animal study was reviewed and approved by the Animal Care and Use Committee of Shantou University Medical College.

## Author Contributions

HZ, QH, and HX designed the study. HZ and ZY conducted the experiments and collected the data. HX interpreted the results and drafted the manuscript. All authors have read and approved the final version of the submitted manuscript.

## Conflict of Interest

The authors declare that the research was conducted in the absence of any commercial or financial relationships that could be construed as a potential conflict of interest.
